# Atorvastatin-associated neurological adverse events: a disproportionality analysis with adjustment for confounding by indication

**DOI:** 10.3389/fmed.2026.1852942

**Published:** 2026-07-07

**Authors:** Xinjun He, Qi Zhou, Min Zhang, Dandan Wang, Xinchang Xu

**Affiliations:** Department of Pharmacy, Hangzhou Third People’s Hospital, Hangzhou, China

**Keywords:** atorvastatin, disproportionality analysis, FAERS database, nervous system diseases, pharmacovigilance

## Abstract

**Background:**

Atorvastatin, a widely prescribed HMG-CoA reductase inhibitor, is associated with neurological adverse events (NAEs) beyond its well-known myopathy; however, comprehensive evaluations accounting for confounding by indication remain limited.

**Methods:**

A disproportionality analysis was performed using the FDA Adverse Event Reporting System (FAERS) database (Q1 2004–Q3 2025) with three signal detection algorithms (PRR, BCPNN, and MGPS). Signal criteria were: PRR ≥ 2, χ^2^ ≥ 4, EBGM_05_ > 2, IC_025_ > 0. To minimize confounding by indication, reports with indications for hypercholesterolemia or coronary artery disease (i.e., the approved label indications of atorvastatin) were excluded.

**Results:**

Of 50 NAE signals identified for atorvastatin, 32 remained significant after exclusion. The strongest signals clustered into three clinically meaningful categories: neuromuscular junction disorders (myasthenia gravis: PRR = 5.65, χ^2^ = 748.22, EBGM_05_ = 4.82), peripheral neuropathies (axonal neuropathy: PRR = 6.15, χ^2^ = 117.78, EBGM_05_ = 4.14; peripheral sensory neuropathy: PRR = 3.42, χ^2^ = 182.34, EBGM_05_ = 2.80), and central nervous system and cognitive disorders (encephalitis toxic: PRR = 7.76, χ^2^ = 17.11, EBGM_05_ = 2.39; brain fog: PRR = 2.94, χ^2^ = 168.76, EBGM_05_ = 2.46). Additionally, exceptionally strong signals without detailed published case support included acute necrotising myelitis (PRR = 832.92, EBGM_05_ = 38.61), vibration syndrome (PRR = 37.02, EBGM_05_ = 14.51), and radiculitis brachial (PRR = 26.03, EBGM_05_ = 13.57).

**Conclusion:**

This study identified multiple strong neurological safety signals associated with atorvastatin using a rigorous disproportionality analysis with adjustment for confounding by indication. These findings provide valuable real-world evidence to guide clinical monitoring and warrant further validation.

## Introduction

Atorvastatin, a 3-hydroxy-3-methylglutaryl-coenzyme A (HMG-CoA) reductase inhibitor, is one of the most commonly prescribed statins for the treatment of hyperlipidemia, coronary artery disease, and other atherosclerotic conditions (1). Despite its well-established cardiovascular benefits (2), the clinical use of atorvastatin is limited by various adverse effects, including myopathy, myalgia, cognitive impairment, and liver dysfunction (3). Recently, Attardo et al. (4) systematically reviewed the neuromuscular adverse effects of statins, highlighting the need for heightened clinical awareness. Recent large-scale pharmacovigilance studies have consistently identified neurological adverse events as a notable safety concern for statin therapy (5, 6).

Although individual case reports and pharmacovigilance studies suggest an association between atorvastatin and neurological adverse events (NAEs), there is currently a lack of comprehensive and up-to-date descriptions of the scope and severity of these events. Moreover, previous analyses may not have adequately accounted for confounding by indication, as hyperlipidemia itself has been independently associated with neurological manifestations (7). For instance, hyperlipidemia has been causally linked to an increased risk of Alzheimer’s disease (8), highlighting the need to distinguish drug effects from those of the underlying condition. Additionally, while several real-world studies have investigated atorvastatin-associated NAEs, none have integrated quantitative pharmacovigilance signals with qualitative case-level evidence, and none have rigorously controlled for confounding by indication. Therefore, a systematic and rigorous assessment of the neurological safety profile of atorvastatin is warranted. The FDA Adverse Event Reporting System (FAERS) is a spontaneous reporting database that collects adverse event reports from healthcare professionals, patients, and manufacturers, enabling post-marketing safety surveillance.

In this study, we aimed to comprehensively investigate the association between atorvastatin and NAEs. We conducted a disproportionality analysis of the FAERS database covering Q1 2004 to Q3 2025. To minimize confounding by indication, we performed stratified analyses by excluding reports where the primary indication for atorvastatin use was hypercholesterolemia or coronary artery disease (i.e., the approved label indications). Multiple signal detection algorithms—including Proportional Reporting Ratio (PRR), Bayesian Confidence Propagation Neural Network (BCPNN) and Multi-Item Gamma Poisson Shrinker (MGPS)—were employed to identify and quantify potential neurological risk signals associated with atorvastatin. The quantitative findings are discussed with reference to published case reports to aid clinical interpretation.

## Methods

### Data source and extraction

A retrospective pharmacovigilance analysis was conducted using the U.S. Food and Drug Administration Adverse Event Reporting System (FAERS) from 2004Q1 to 2025Q3. Demographic characteristics and reporting status of patients experiencing neurological adverse events associated with atorvastatin were analyzed. We identified all preferred terms (PTs) for NAEs classified under the System Organ Class (SOC) “nervous system disorders.” Each report in the pharmacovigilance database was coded by PT, corresponding to all adverse events from the Medical Dictionary for Regulatory Activities MedDRA (version 25.1). We extracted the following data from FAERS: the number of NAEs caused by atorvastatin, the total number of adverse events caused by atorvastatin, the total number of NAEs caused by all drugs, and the total number of adverse events reported to FAERS.

### Data preprocessing and deduplication

Before disproportionality analysis, the FAERS data were preprocessed to ensure data quality and replicability. First, duplicate reports were removed by retaining the most recent FDA_DT (receipt date) for each unique CASEID. Second, only reports where atorvastatin was coded as the primary suspect (PS) or secondary suspect (SS) were included; reports with atorvastatin as concomitant or interacting drug were excluded. Third, missing data for age or sex were coded as “unknown” and retained in the analysis. The deduplication procedure followed the method described by Sakaeda et al. (9) and Noguchi et al. (10).

### Disproportionality analysis of spontaneous reports

To enhance the reliability of drug-adverse event association analysis, a combined strategy of disproportionality analysis and Bayesian methods was employed to systematically identify signals of NAEs associated with atorvastatin. The strength of drug-event associations was quantified from two perspectives by simultaneously calculating four metrics: Proportional Reporting Ratio (PRR) with chi-square (χ^2^), Bayesian Confidence Propagation Neural Network (BCPNN), and Multi-Gamma Poisson Shrinker (MGPS). The definitions of a, b, c, d, and *n* used in the calculations are presented in Table 1. The formulas for these algorithms are listed in Table 2. Higher values from each algorithm indicate stronger association signals. A signal was deemed present when the following criteria were met: co-occurrence frequency ≥ 3.0, PRR ≥ 2.0, with a chi-square (χ^2^) value ≥4.0, the lower limit of 95% CI of empirical Bayesian geometric mean (EBGM_05_) > 2, and the lower limit of 95% CI of the information component (IC_025_) > 0. If all outcome measures satisfy the aforementioned thresholds, the signal was confirmed. All analyses were conducted using spontaneous reporting data processed with R software (version 4.3.3) and the following packages: dplyr, tidyr, and PhViD, as well as Microsoft Excel.

**Table 1:** 2 × 2 contingency table for disproportionality analysis.

Drug	Target NAEs	Non-target NAEs	Total
Atorvastatin	a	b	a + b
Non-atorvastatin	c	d	c + d
Total	a + c	b + d	a + b + c + d

NAEs, neurological adverse events; a, number of reports containing both the target drug and target NAEs; b, number of reports containing the target drug and non-target NAEs; c, number of reports containing other drugs and target NAEs; d, number of reports containing other drugs and non-target NAEs.

**Table 2:** Three major algorithms used for signal detection.

Algorithms	Equation	Criteria
PRR	PRR = a(c + d)/c/(a + b)	a ≥ 3, PRR ≥ 2
PRR χ^2^	χ^2^ = (a + b + c + d)[(ad − bc)^∧2^]/[(a + b) (c + d) (a + c) (b + d)]	χ^2^ ≥ 4
BCPNN	IC = log_2_[a(a + b + c + d)/((a + c)/(a + b))]	
BCPNN 95%CI	95% CI = E(IC) ± 2 × √(V(IC))	IC_025_ > 0
MGPS	EBGM = (a × (a + b + c + d))/[(a + c)/(a + b)]	
MGPS 95%CI	95% CI = e^(ln(EBGM) ± 1.96 × √(1/a + 1/b + 1/c + 1/d))	EBGM_05_ > 2

The meanings of a, b, c, d can be seen in Table 1; 95% CI, 95% confidence interval; χ2, chi-squared; IC, information component; IC_025_, the lower limit of 95% CI, of the IC; E(IC), the IC, expectations; V(IC), the variance of IC; EBGM, empirical Bayesian geometric mean; EBGM_05_, the lower limit of 95% CI of EBGM.

To partially adjust for confounding by indication, we performed a sensitivity analysis by excluding reports whose reported indication (INDI_PT) corresponded to the approved label indications of atorvastatin, i.e., hypercholesterolemia and coronary artery disease. Specifically, we excluded reports where INDI_PT was any of the following terms: “hyperlipidemia”, “hypercholesterolemia”, “dyslipidemia”, “low density lipoprotein increased”, “type IIa hyperlipidaemia”, “blood cholesterol increased”, “blood cholesterol”, “blood triglycerides increased”, “atherosclerosis”, “coronary artery disease”, and “myocardial infarction”. Other indications (including hypertension, diabetes mellitus, and cerebrovascular diseases) were not excluded, as the goal was to remove the most direct sources of confounding (lipid disorders and established coronary disease) while retaining a clinically representative sample. After excluding these reports, we recalculated the PRR, χ^2^, EBGM_05_, and IC_025_ for each neurological adverse event to assess the robustness of the identified signals.

## Results

### Disproportionality analysis

As of Q3 2025, the FAERS database recorded a total of 30,459 reports of NAEs associated with atorvastatin. Among affected patients, 50.0% were female, 43.0% were male, and 7.0% had missing gender data.

Through disproportionality analysis, a total of 50 significant NAE signals were identified for atorvastatin. After excluding reports with indications for hypercholesterolemia or coronary artery disease (the approved label indications of atorvastatin), 32 of the 50 signals remained significant. Notably, signals such as Alzheimer’s dementia and senile dementia—which had shown moderate strength in the overall analysis—were no longer present after this exclusion, suggesting these associations may be partly driven by confounding by indication rather than a direct drug effect.

The most robust signals clustered into three clinically meaningful categories: Neuromuscular junction disorders emerged as the strongest and most consistent signal category. Myasthenia gravis showed substantial signal strength (unadjusted PRR = 5.65; χ^2^ = 748.22; EBGM_05_ = 4.82; IC_025_ = 2.23), which persisted and became even stronger after exclusion (adjusted PRR = 7.73; χ^2^ = 505.08; EBGM_05_ = 6.21; IC_025_ = 2.52). Myasthenia gravis crisis also demonstrated a strong signal that was markedly enhanced after exclusion (adjusted PRR = 11.10; χ^2^ = 199.36; EBGM_05_ = 7.19; IC_025_ = 2.33). Both signals remained significant, indicating robust drug-event relationships.

Peripheral neuropathies constituted another well-supported signal category. Peripheral sensory neuropathy (PRR = 3.42; χ^2^ = 182.34; EBGM_05_ = 2.80; IC_025_ = 1.45) and axonal neuropathy (PRR = 6.15; χ^2^ = 117.78; EBGM_05_ = 4.14; IC_025_ = 1.82) demonstrated strong associations that remained significant after exclusion, with adjusted estimates showing enhancement (PRR = 8.35 and 8.98, respectively).

Central nervous system and cognitive disorders also yielded notable signals. Encephalitis toxic (PRR = 7.76; χ^2^ = 17.11; EBGM_05_ = 2.39; IC_025_ = 0.04) and brain fog (PRR = 2.94; χ^2^ = 168.76; EBGM_05_ = 2.46; IC_025_ = 1.27) demonstrated strong associations that remained significant after exclusion.

In addition to the three well-corroborated categories described above, several other signals exhibited exceptionally strong disproportionality that did not correspond to published case reports. Acute necrotising myelitis (PRR = 832.92; χ^2^ = 1292.54; EBGM_05_ = 38.61; IC_025_ = 1.57), vibration syndrome (PRR = 37.02; χ^2^ = 212.29; EBGM_05_ = 14.51; IC_025_ = 1.62), and radiculitis brachial (PRR = 26.03; χ^2^ = 303.72; EBGM_05_ = 13.57; IC_025_ = 2.45) represented the most prominent signals, with disproportionality levels substantially exceeding those in the categorized signals. Additional notable signals included quadriparesis (PRR = 12.48; χ^2^ = 1053.71; EBGM_05_ = 9.79; IC_025_ = 3.14), hemihypoaesthesia (PRR = 10.46; χ^2^ = 98.35; EBGM_05_ = 5.64; IC_025_ = 1.75), and myotonia (PRR = 10.27; χ^2^ = 88.19; EBGM_05_ = 5.40; IC_025_ = 1.65). All of these signals remained significant after exclusion, with adjusted estimates showing further enhancement for most. However, for transient aphasia, the adjusted analysis yielded zero reports, precluding calculation of post-exclusion signal metrics; therefore, the strength of this association after exclusion of label indications remains uncertain. The detailed results of the disproportionality analysis are presented in Table 3.

**Table 3:** Results of disproportionality analysis of neurological adverse event reports for atorvastatin identified based on the FAERS database.

PT	Unadjusted				Adjusted			
	PRR	χ^2^	EBGM_05_	IC_025_	PRR	χ^2^	EBGM_05_	IC_025_
Acute necrotising myelitis	**832.92**	**1292.54**	**38.61**	**1.57**	**2655.23**	**4127.24**	**122.73**	**1.61**
Capsular warning syndrome	**73.22**	**217.90**	**18.33**	**0.76**	**233.43**	**707.91**	**58.28**	**0.83**
Vibration syndrome	**37.02**	**212.29**	**14.51**	**1.62**	**118.01**	**702.81**	**46.11**	**1.81**
Progressive muscular atrophy	27.20	90.58	8.71	0.73	—	—	—	—
Radiculitis brachial	**26.03**	**303.72**	**13.57**	**2.45**	**76.45**	**879.42**	**39.31**	**2.75**
Peripheral nerve palsy	20.54	205.39	10.53	2.16	—	—	—	—
Bulbar palsy	**17.00**	**295.12**	**10.23**	**2.61**	**9.76**	**31.04**	**3.60**	**0.52**
Polyneuropathy idiopathic progressive	14.34	58.50	5.51	0.91	—	—	—	—
Transient aphasia	13.92	113.26	6.98	1.75	—	—	—	—
Transient global amnesia	**13.85**	**608.29**	**9.99**	**3.03**	**8.59**	**72.99**	**4.70**	**1.55**
Lower motor neurone lesion	13.73	33.48	4.07	0.20	—	—	—	—
Amyotrophic lateral sclerosis	**12.87**	**1401.69**	**10.31**	**3.25**	**6.98**	**121.87**	**4.63**	**1.90**
Quadriparesis	**12.48**	**1053.71**	**9.79**	**3.14**	**24.53**	**1443.18**	**18.62**	**3.79**
Quadrantanopia	11.44	90.91	5.81	1.63	—	—	—	—
Hemihypoaesthesia	**10.46**	**98.35**	**5.64**	**1.75**	**33.35**	**360.67**	**17.94**	**2.42**
Myotonia	**10.27**	**88.19**	**5.40**	**1.65**	**26.57**	**213.95**	**13.22**	**1.96**
Motor neurone disease	9.76	181.33	6.26	2.23	—	—	—	—
Neuromyopathy	**9.29**	**370.25**	**6.81**	**2.56**	**22.00**	**759.67**	**15.57**	**3.36**
Diabetic neuropathy	8.93	1595.30	7.59	2.87	—	—	—	—
Encephalitis toxic	**7.76**	**17.11**	**2.39**	**0.04**	**24.74**	**66.18**	**7.60**	**0.36**
Piriformis syndrome	7.14	30.75	3.09	0.80	—	—	—	—
Carotid arteriosclerosis	6.78	182.20	4.80	2.06	—	—	—	—
Axonal neuropathy	**6.15**	**117.78**	**4.14**	**1.82**	**8.98**	**91.14**	**5.14**	**1.73**
Sensory overload	5.95	20.09	2.40	0.49	—	—	—	—
Cerebral microangiopathy	5.95	28.12	2.75	0.82	—	—	—	—
Myasthenia gravis	**5.65**	**748.22**	**4.82**	**2.23**	**7.73**	**505.08**	**6.21**	**2.52**
Neurologic neglect syndrome	**5.47**	**57.14**	**3.27**	**1.39**	**6.45**	**27.38**	**2.87**	**0.76**
Paresis	**5.18**	**207.91**	**3.97**	**1.89**	**10.40**	**335.24**	**7.52**	**2.61**
Carotid artery occlusion	**4.88**	**277.89**	**3.90**	**1.90**	**3.16**	**27.93**	**2.01**	**0.86**
Senile dementia	4.81	53.30	2.97	1.31	—	—	—	—
Vocal cord paresis	**4.81**	**17.74**	**2.11**	**0.52**	**15.33**	**78.76**	**6.70**	**1.22**
Loss of proprioception	**4.69**	**22.77**	**2.29**	**0.75**	**9.27**	**36.46**	**3.80**	**0.77**
Amnesia	**4.20**	**3598.46**	**3.94**	**1.97**	**2.33**	**201.73**	**2.07**	**1.04**
Myasthenia gravis crisis	**4.13**	**60.57**	**2.76**	**1.31**	**11.10**	**199.36**	**7.19**	**2.33**
Demyelination	**4.06**	**199.76**	**3.25**	**1.65**	**10.56**	**614.84**	**8.27**	**2.87**
Dementia Alzheimer’s type	3.86	412.60	3.31	1.70	—	—	—	—
Cranial Nerve Disorder	3.50	24.59	2.04	0.82	—	—	—	—
Essential tremor	**3.48**	**34.91**	**2.22**	**0.99**	**4.40**	**20.93**	**2.19**	**0.71**
Peripheral sensory neuropathy	**3.42**	**182.34**	**2.80**	**1.45**	**8.35**	**531.28**	**6.66**	**2.61**
Paralysis	**3.33**	**424.54**	**2.92**	**1.53**	**3.87**	**205.11**	**3.15**	**1.61**
Polyneuropathy	**3.31**	**330.88**	**2.86**	**1.50**	**2.81**	**65.19**	**2.16**	**1.06**
Demyelinating polyneuropathy	**3.28**	**28.22**	**2.04**	**0.87**	**4.62**	**22.55**	**2.29**	**0.75**
Carotid artery stenosis	**3.22**	**87.73**	**2.46**	**1.24**	**5.46**	**112.03**	**3.81**	**1.74**
Muscle contractions involuntary	3.19	92.15	2.46	1.25	—	—	—	—
Monoparesis	**3.17**	**45.53**	**2.21**	**1.05**	**7.81**	**141.01**	**5.18**	**2.03**
Brain fog	**2.94**	**168.76**	**2.46**	**1.27**	**5.97**	**349.23**	**4.79**	**2.18**
Paraparesis	**2.93**	**36.50**	**2.02**	**0.92**	**4.49**	**37.69**	**2.64**	**1.11**
Sensory loss	2.79	140.68	2.32	1.19	—	—	—	—
Dysstasia	**2.75**	**493.99**	**2.48**	**1.30**	**2.53**	**120.58**	**2.12**	**1.07**
Neuropathy peripheral	2.66	1401.16	2.50	1.32	—	—	—	—

Bold font represents positive adverse event signals satisfying the three signal criteria. PRR, Proportional Reporting Ratio; χ^2^, Chi-square value; EBGM₀₅, Lower bound of empirical Bayesian geometric mean; IC₀₂₅, Lower bound of information component. The adjusted results were obtained from sensitivity analysis by excluding reports involving primary indications (INDI_PT). Em dashes (—) indicate no valid reports remained after exclusion.

## Discussion

This study analyzed postmarketing data from the FAERS pharmacovigilance database to evaluate the potential risk of NAEs associated with atorvastatin use in clinical practice. Through disproportionality analysis, we identified 50 positive signals of NAEs, of which 32 remained significant after exclusion of label indications to minimize confounding by indication. It is important to note that FAERS data can only generate hypothesis-generating signals and cannot establish causality; the findings should be interpreted as suggestive rather than confirmatory. The findings provide a robust, real-world quantification of the neurological safety profile of atorvastatin.

The most robust signals clustered into multiple clinically meaningful categories. Neuromuscular junction disorders emerged as the strongest and most consistent signal category. Myasthenia gravis (PRR = 5.65; χ^2^ = 748.22; EBGM_05_ = 4.82) suggested substantial signal strength that persisted after exclusion of label indications. This statistical finding is supported by published case reports documenting positive anti-acetylcholine receptor antibodies, electrophysiological evidence of neuromuscular transmission defects, and clinical improvement following drug discontinuation (11–17). Such case-level evidence supports the plausibility of a causal relationship.

Peripheral neuropathies constituted another well-supported signal category. Axonal neuropathy (PRR = 6.15) and peripheral sensory neuropathy (PRR = 3.42) indicated strong associations, with previous case reports providing pathological confirmation through skin biopsy showing reduced epidermal nerve fiber density and nerve conduction studies revealing axonal polyneuropathy (18–20). These clinical observations align with the quantitative signals.

Central nervous system and cognitive disorders also yielded notable signals. Encephalitis toxic (PRR = 7.76) and brain fog (PRR = 2.94) showed strong associations that remained significant after exclusion of label indications. In contrast, the signal for senile dementia disappeared after exclusion of label indications, suggesting confounding by indication. Previously reported cases of reversible cognitive impairment, memory deficits, and disorientation following atorvastatin use (21–26) provide clinical context for the brain fog and encephalitis toxic signals. Additionally, hemihypoaesthesia (PRR = 10.46) suggested strong disproportionality, indicating possible central sensory pathway involvement, though no case reports have specifically described this phenomenon.

In addition to these clinically characterized events, several signals demonstrated exceptionally strong disproportionality but lack detailed description in available case reports. These included acute necrotising myelitis (PRR = 832.92; EBGM_05_ = 38.61), vibration syndrome (PRR = 37.02; EBGM_05_ = 14.51), and radiculitis brachial (PRR = 26.03; EBGM_05_ = 13.57). Extremely high disproportionality values (e.g., PRR = 832.92 for acute necrotising myelitis) should be interpreted with caution, as they are likely influenced by very small case numbers and may not reflect true effect sizes. These signals are hypothesis-generating rather than confirmatory. While these events represent statistically prominent signals warranting clinical attention, their rarity and the absence of detailed case reports preclude in-depth clinical characterization. Notably, acute necrotising myelitis represents the strongest signal identified in this study; to date, no published case report or mechanistic study has directly linked atorvastatin to myelitis, and the extremely high disproportionality stands without clinical or mechanistic precedent. It may reflect a true but exceptionally rare adverse event, reporting bias, or unmeasured confounding.

Regarding vibration syndrome, Jones et al. (27) reviewed drug-induced peripheral neuropathy and noted that statins have been implicated in sensory disturbances. West et al. (28) conducted a pilot study on statin use and peripheral sensory perception, finding evidence of altered vibration perception in statin users. Vibration syndrome is typically associated with occupational exposure to prolonged use of handheld vibrating tools. However, the FAERS database does not capture occupational history; therefore, we cannot determine whether patients with this reported event had such exposures. The emergence of this signal may reflect unmeasured confounding by occupational factors rather than a direct drug effect, although a novel statin-associated phenomenon cannot be entirely excluded.

Brachial radiculitis (Parsonage-Turner syndrome) is characterized by acute shoulder pain followed by upper limb weakness; to our knowledge, drug-induced cases have rarely been reported. These signals should be interpreted as hypothesis-generating rather than confirmatory, but their statistical prominence suggests that clinicians should remain alert to potential upper limb neurologic symptoms in atorvastatin-treated patients.

Capsular warning syndrome, a form of transient ischemic attack, also showed a strong signal after exclusion of label indications (adjusted PRR = 233.43). However, because cerebrovascular indications were retained in the adjusted analysis, this signal likely reflects the natural history of underlying cerebrovascular disease rather than a direct drug effect.

The pathophysiological mechanisms underlying atorvastatin-associated myasthenia gravis remain incompletely understood, but several interrelated hypotheses have been proposed (17). Mitochondrial dysfunction represents a primary mechanism: statins inhibit HMG-CoA reductase, reducing coenzyme Q10 synthesis, which impairs mitochondrial respiration and increases oxidative stress (29). These changes may contribute to peripheral axonal degeneration and dysfunction in high-energy-demanding tissues such as skeletal muscle and the neuromuscular junction, consistent with the observed axonal neuropathy patterns and frequent extraocular muscle involvement in published cases. Immune-mediated mechanisms also play a key role. The presence of anti-acetylcholine receptor antibodies in multiple cases suggests statins may trigger or unmask autoimmune processes. Proposed mechanisms include statin-induced upregulation of major histocompatibility complex class II molecules on antigen-presenting cells, enhanced T-cell activation, molecular mimicry involving HMG-CoA reductase, and a shift in T-cell polarization toward TH2 dominance, which may promote antibody production (30, 31). Additionally, lipophilic statins may induce muscle-related adverse events via muscle-specific autophagy (32), and statins may directly influence autoimmunity contributing to muscle involvement. The temporal relationship between drug initiation and symptom onset, together with improvement after discontinuation, supports a drug-induced autoimmune etiology. Recent pharmacovigilance studies have confirmed the association between statins and myasthenia gravis, with atorvastatin being the most frequently reported agent (16). Regulatory agencies have also issued safety alerts regarding this risk, recommending prompt discontinuation when MG symptoms occur.

Direct neurotoxicity may contribute to the development of both axonal neuropathy and peripheral sensory neuropathy. Studies have shown that statins interfere with cholesterol synthesis in Schwann cells; since cholesterol is a key component for maintaining the structure and function of myelin sheaths, this process may disrupt myelin formation and stability (33, 34). Concurrently, statin-induced alterations in small GTPase signaling pathways may affect axonal transport and cytoskeletal integrity (35). Together, these mechanisms constitute the molecular basis of statin-associated neuropathy (36). Clinically, skin biopsy-confirmed findings of burning sensations and small-fiber neuropathy as well as nerve conduction studies confirming axonal pathology, are highly consistent with this mechanistic framework.

The mechanism by which atorvastatin affects cognitive function remains unclear and is subject to ongoing debate. Available clinical evidence is conflicting: some studies suggest it may alleviate memory impairment or delay cognitive decline (37–39), whereas others indicate that patients taking atorvastatin derive no such benefits and may even experience worsening memory (40, 41). One possible mechanism involves cholesterol metabolism. Cholesterol is essential for maintaining neuronal membrane integrity, synaptic function, and myelin formation. Theoretically, statins may reduce cerebral blood flow by inhibiting the synthesis of endogenous vasodilators, or they may directly affect neuronal membrane fluidity and receptor function, thereby leading to changes in cognitive function. Importantly, the rapid reversal of cognitive symptoms following discontinuation of the medication suggests that these changes are primarily functional rather than structural, indicating that they stem from reversible metabolic or signaling disturbances rather than permanent neuronal damage. Published cases of central nervous system symptoms (e.g., encephalitis toxic, brain fog) have shown resolution or improvement after drug discontinuation. The NHANES database analysis further suggests that while atorvastatin may have a protective effect on memory in some populations, individual patients may still experience cognitive side effects (42). A real-world FAERS analysis specifically identified statin-related neurocognitive disorder as a notable safety concern (43).

This study has several limitations. First, the FAERS database is a spontaneous reporting system, which is subject to underreporting, reporting bias (e.g., Weber effect), and the absence of denominator data for calculating incidence rates. Second, while we excluded reports with label indications (hypercholesterolemia and coronary artery disease) to minimize confounding by indication, other potential confounders such as concomitant medications, comorbidities, and patient demographics could not be fully controlled. Furthermore, residual confounding may remain from other indications (e.g., hypertension, diabetes mellitus, cerebrovascular disease) that were not excluded. A more stringent analysis excluding all cardiovascular and cerebrovascular indications would require a much larger dataset and is beyond the scope of this study. Third, the lack of detailed clinical information in FAERS records (e.g., dosage, duration of therapy, rechallenge data) limits our ability to assess dose–response relationships or confirm causality. Fourth, the small number of published case reports for certain rare events limits the generalizability of clinical correlations; however, the quantitative signals themselves remain valid as hypothesis-generating evidence. Fifth, our sensitivity analysis only excluded indications directly corresponding to hypercholesterolemia and coronary artery disease. Indications such as hypertension, diabetes mellitus, and cerebrovascular disease were retained in the adjusted analysis. Therefore, residual confounding by these conditions remains possible, particularly for neurological events known to be associated with vascular risk factors (e.g., cognitive impairment, stroke). A more stringent analysis excluding all cardiovascular and cerebrovascular indications would require a much larger dataset and is beyond the scope of this study. Nonetheless, the persistence of signals such as myasthenia gravis and axonal neuropathy after removing the primary label indications supports a genuine drug–event association that is unlikely to be explained solely by confounding by indication.

Despite these limitations, the findings of this study have several practical implications for clinical practice. First, baseline neurological assessment should be considered before initiating atorvastatin, particularly in patients with pre-existing neuromuscular disorders or those taking medications that affect neuromuscular transmission. Second, patients should be counseled about potential neurological symptoms including unexplained muscle weakness, sensory disturbances, word-finding difficulties, or mood changes. Third, given that most documented cases show improvement after drug withdrawal, prompt discontinuation should be considered when NAEs occur. Fourth, because the time to onset can vary from days to years, vigilance should be maintained throughout the treatment course, not only during the initial months of therapy.

## Conclusion

This study identified multiple important neurological safety signals associated with atorvastatin through a disproportionality analysis of the FAERS database, with a substantial proportion of signals remaining significant after excluding reports with indications for hypercholesterolemia or coronary artery disease. Furthermore, the most robust signals included neuromuscular junction disorders (myasthenia gravis), peripheral neuropathies (axonal and peripheral sensory neuropathy), and central nervous system disorders (encephalitis toxic and brain fog). In addition, signals such as acute necrotising myelitis demonstrated strong disproportionality but warrant further clinical validation. Therefore, clinicians should remain vigilant for NAEs throughout atorvastatin therapy, as onset can range from days to years after treatment initiation. Nevertheless, these findings serve as an important reminder for clinicians to monitor for potential neurological adverse events during atorvastatin therapy, while recognizing that FAERS-based signals are hypothesis-generating and require confirmation through other study designs.

## Data Availability

The raw data supporting the conclusions of this article will be made available by the authors, without undue reservation.
